# A randomized phase 1/2a trial of ExPEC10V vaccine in adults with a history of UTI

**DOI:** 10.1038/s41541-024-00885-1

**Published:** 2024-06-14

**Authors:** Carlos A. Fierro, Michal Sarnecki, Bart Spiessens, Oscar Go, Tracey A. Day, Todd A. Davies, Germie van den Dobbelsteen, Jan Poolman, Darren Abbanat, Wouter Haazen

**Affiliations:** 1 Johnson County Clin-Trials, Lenexa, KS USA; 2 Janssen Research & Development, Infectious Diseases & Vaccines, Janssen Vaccines, Bern, Switzerland; 3 Janssen Research & Development, Infectious Diseases & Vaccines, Janssen Pharmaceutica, Beerse, Belgium; 4 Janssen Research & Development, Raritan, NJ USA; 5 Bacterial Vaccines Discovery and Early Development, Janssen Vaccines & Prevention B.V., Leiden, Netherlands

**Keywords:** Conjugate vaccines, Bacterial infection, Infectious-disease diagnostics

## Abstract

The safety, reactogenicity, and immunogenicity of 3 doses of ExPEC10V (VAC52416), a vaccine candidate to prevent invasive *Escherichia coli* disease, were assessed in a phase 1/2a study (NCT03819049). In Cohort 1, ExPEC10V was well tolerated; the high dose was selected as optimal and further characterized in Cohort 2. Cohort 2 comprised a maximum 28-day screening, vaccination (Day 1), double-blind 181-day follow-up, and open-label long-term follow-up until Year 1. Healthy participants (≥60 years) with a history of urinary tract infection (UTI) within 5 years were randomized to receive ExPEC10V or placebo. The primary endpoint evaluated the safety and reactogenicity of ExPEC10V (solicited local and systemic AEs [until Day 15]; unsolicited AEs [until Day 30], SAEs [until Day 181], and immunogenicity [Day 30]) via multiplex electrochemiluminescent (ECL) and multiplex opsonophagocytic assay (MOPA). 416 participants (ExPEC10V, *n* = 278; placebo, *n* = 138) were included (mean age [SD], 68.8 [6.52] years; female, 79.6%; White, 96.1%). The incidence of solicited AEs was higher with ExPEC10V (local, 50.0% [*n* = 139]; systemic, 50.0% [*n* = 139]) than placebo (15.9% [*n* = 22]; 38.4% [*n* = 53]); rates of unsolicited AEs were comparable (ExPEC10V, 28.4% [*n* = 79]; placebo, 26.1% [*n* = 36]). No vaccine-related SAEs or deaths were reported. ExPEC10V elicited a robust antibody-mediated immunogenic response across all serotypes with ECL (Day 30 geometric mean fold increase, 2.33–8.18) and demonstrated functional opsonophagocytic killing activity across all measured serotypes (Day 30 geometric mean fold increase, 1.81–9.68). ExPEC10V exhibited an acceptable safety profile and a robust vaccine-induced functional immunogenic response in participants with a history of UTI. Clinical trial registration details: https://clinicaltrials.gov/study/NCT03819049.

## Introduction

*Escherichia coli* is a ubiquitous gram-negative bacterial species with a wide range of diverse genetic lineages that can cause pathogenic disease^1,2^. Extraintestinal pathogenic *E. coli* (ExPEC) is the most common Gram-negative bacterial pathogen in humans^3^. ExPEC infection of normally sterile parts of the body, known as invasive *E. coli* disease (IED), can progress to sepsis, septic shock, or death if treated too late or improperly^3–5^. Clinically, IED can be defined as a bacterial infection with acute systemic consequences, including bacteremia and sepsis, based on clinical criteria and microbiological confirmation by the isolation and identification of *E. coli* from blood or other normally sterile body sites as well as from urine in patients with urosepsis and no other identifiable source of infection^1,6^.

Both the spectrum and the frequency of antimicrobial-resistant *E. coli* are escalating rapidly^6–10^, and in parallel, steady increases in rates of *E. coli* bacteremia and sepsis have been observed^4,11–13^. Globally, more than a quarter of all reported bacteremia cases are caused by *E. coli*^4^. While *E. coli* has been reported as the most common cause of hospital-associated disease in the United States^14,15^, the epidemiology may be shifting toward community-onset infections, with 33–58% of identified IED cases acquired in a community setting in recent epidemiological reports from the United Kingdom and Australia, respectively^8,13^. Unsurprisingly, there are significant individual, institutional, and societal costs associated with IED with a high economic burden resulting from outpatient medical visits and hospitalizations^4,16^. The estimated IED fatality rate is between 12 and 20%^4,5,17^.

Individuals with a history of urinary tract infection (UTI), as well as older individuals, may be at higher risk for IED. *E. coli* is responsible for 75–95% of UTIs in both community and healthcare settings^18–20^. Approximately half of all IED cases have a urinary origin^5,21,22^. Further, incidence of IED is consistently reported to be higher in individuals ≥65 years, and the increase is even more pronounced in those ≥85 years^4,21,22^. Preventive measures to combat IED could provide a substantial clinical benefit to these high-risk populations.

ExPEC10V is a 10-valent *E. coli* bioconjugate vaccine candidate for the prevention of IED caused by 10 O-serotypes most frequently associated with invasive *E. coli* infections^23,24^. The vaccine contains the O-antigen polysaccharides of ExPEC serotypes O1A, O2, O4, O6A, O8, O15, O16, O18A, O25B, and O75. This was a 2-cohort, phase 1/2a study (VAC52416BAC1001, NCT03819049) of the safety, reactogenicity, and immunogenicity of ExPEC10V in healthy adults aged ≥60 years. Cohort 1 revealed a strong safety profile and robust immunogenic response to 3 doses of ExPEC10V. The high dose was selected as optimal^24^. Here, results from Cohort 2 are described, which aimed to further characterize the short- and long-term safety and immunogenicity of the selected dose of ExPEC10V in healthy adults aged ≥60 years with a history of UTI.

## Results

### Participants

**Fig. 1:**
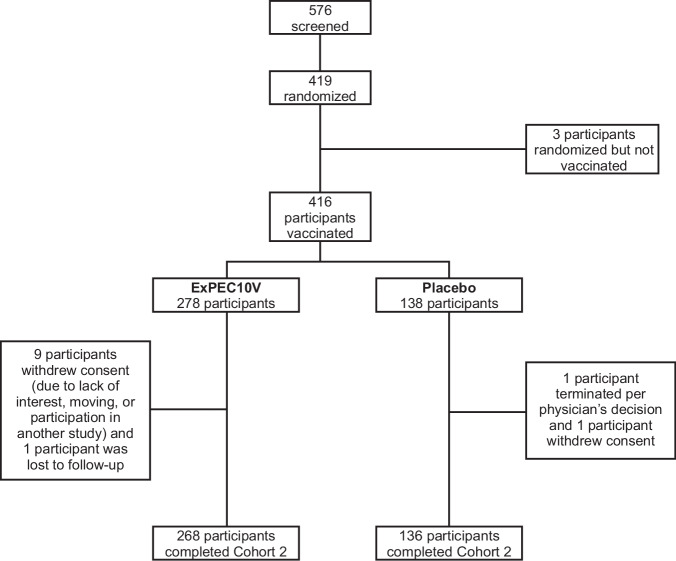
Study disposition for cohort 2 participants.

A total of 576 participants were screened for the study; 419 met the eligibility criteria, and 416 were randomized and vaccinated (Fig. 1); 278 participants were randomly assigned to receive ExPEC10V, and 138 participants were randomly assigned to receive placebo. A total of 404 participants (97.1%) completed the study. Of the 12 participants (2.9%) who discontinued the study, 10 withdrew consent (ExPEC10V, 9; placebo, 1), 1 (ExPEC10V) was lost to follow-up, and 1 (placebo) discontinued the study per physician decision. Mean (SD) age was 68.8 (6.52) years, with 80.0% (*n* = 333) of participants between the ages of 60 and 74 years and 20.0% (*n* = 83) ≥75 years at baseline. Most participants were female (79.6% [*n* = 331]), White (96.1% [*n* = 390]), and of non-Hispanic/non-Latino ethnicity (85.1% [*n* = 354]) (Table 1). Participant demographic characteristics were balanced across treatment groups. Concomitant therapies were reported in 90.6% (*n* = 377) of participants, with the most commonly used agents being paracetamol (18.8%), acetylsalicylic acid (16.8%), atorvastatin (15.9%), omeprazole (14.7%), cholecalciferol (11.5%), and metformin (10.6%).

**Table 1:** Summary of participant demographics and baseline characteristics

	ExPEC10V	Placebo	Total
*N*	278	138	416
Age, mean (SD), years	68.7 (6.16)	69.1 (7.21)	68.8 (6.52)
Range	(60–88)	(60–86)	(60–88)
60–64 years (%)	90 (32.4)	48 (34.8)	138 (33.2)
65–69 years (%)	69 (24.8)	30 (21.7)	99 (23.8)
70–74 years (%)	71 (25.5)	25 (18.1)	96 (23.1)
≥75 years (%)	48 (17.3)	35 (25.4)	83 (20.0)
Sex			
Female (%)	220 (79.1)	111 (80.4)	331 (79.6)
Male (%)	58 (20.9)	27 (19.6)	85 (20.4)
Race			
White (%)	261 (96.0)	129 (96.3)	390 (96.1)
Black/African American (%)	7 (2.6)	4 (3.0)	11 (2.7)
Asian (%)	1 (0.4)	1 (0.7)	2 (0.5)
American Indian/Alaska Native (%)	1 (0.4)	0	1 (0.2)
Multiple (%)	2 (0.7)	0	2 (0.5)
Ethnicity			
Not Hispanic or Latino (%)	237 (85.3)	117 (84.8)	354 (85.1)
Hispanic or Latino (%)	38 (13.7)	17 (12.3)	55 (13.2)
Not reported (%)	3 (1.1)	4 (2.9)	7 (1.7)
Body mass index, mean (SD), kg/m^2^	28.1 (4.60)	27.6 (4.65)	27.9 (4.61)
Range	(19–39)	(19–38)	(19–39)

Data presented are *n* (%) unless otherwise specified. Total number of participants with non-missing data used as denominator. Data from the full analysis set (FAS) are presented. The FAS included all randomized participants with a vaccine administration documented and was the primary safety population.

*SD* standard deviation.

### Safety and reactogenicity

**Fig. 2:**
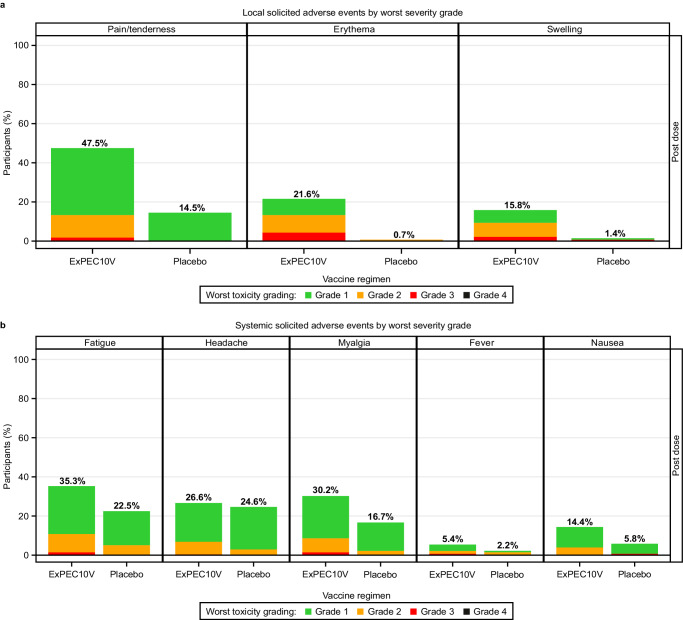
Solicited local and systemic adverse events by worst severity grad. **a** Local solicited adverse events by worst severity grade and **b** Systemic solicited adverse events by worst severity grade.

The incidence of solicited local and systemic AEs was higher with ExPEC10V than with placebo (Table 2). Solicited AEs occurred in 64.4% of ExPEC10V participants and 42.8% of placebo participants. Solicited local AEs were observed in 50.0% of ExPEC10V participants and 15.9% of placebo participants. The most frequently solicited local AE was pain/tenderness, reported by 47.5% of ExPEC10V participants and 14.5% of placebo participants. Solicited systemic AEs were observed in 50.0% of ExPEC10V participants and 38.4% of placebo participants. The most frequently solicited systemic AEs were fatigue (ExPEC10V, 35.3%; placebo, 22.5%), myalgia (ExPEC10V, 30.2%; placebo, 16.7%), and headache (ExPEC10V, 26.6%; placebo, 24.6%). Most solicited AEs were grade 1 in severity (Fig. 2). Grade 3 solicited local and systemic AEs were reported by 6.8% and 3.2% of ExPEC10V participants and 0.7% and 0.7% of placebo participants, respectively. No grade 4 events occurred.

**Table 2:** Summary of adverse events catching

Study vaccination group	ExPEC10V (%)	Placebo (%)
*N*	278	138
Solicited AEs	179 (64.4)	59 (42.8)
Solicited AEs of grade 3	25 (9.0)	2 (1.4)
Solicited local AEs	139 (50.0)	22 (15.9)
Pain/tenderness	132 (47.5)	20 (14.5)
Erythema	60 (21.6)	1 (0.7)
Swelling	44 (15.8)	2 (1.4)
Solicited local AEs of grade 3	19 (6.8)	1 (0.7)
Solicited systemic AEs	139 (50.0)	53 (38.4)
Fatigue	98 (35.3)	31 (22.5)
Headache	74 (26.6)	34 (24.6)
Myalgia	84 (30.2)	23 (16.7)
Fever	15 (5.4)	3 (2.2)
Nausea	40 (14.4)	8 (5.8)
Solicited systemic AEs of grade 3	9 (3.2)	1 (0.7)
Solicited systemic AEs related to study vaccine	118 (42.4)	41 (29.7)
Solicited systemic AEs of grade 3 related to study vaccine	8 (2.9)	1 (0.7)
Any unsolicited AEs^a^	79 (28.4)	36 (26.1)
Urinary tract infection	7 (2.5)	10 (7.2)
COVID-19	3 (1.1)	1 (0.7)
Cystitis	3 (1.1)	1 (0.7)
Myalgia	7 (2.5)	6 (4.3)
Back pain	3 (1.1)	1 (0.7)
Arthralgia	3 (1.1)	0
Fatigue	5 (1.8)	2 (1.4)
Injection-site pruritis	7 (2.5)	0
Diarrhea	3 (1.1)	3 (2.2)
Nausea	4 (1.4)	0
Headache	5 (1.8)	3 (2.2)
Vertigo	3 (1.1)	1 (0.7)
Oropharyngeal pain	1 (0.4)	2 (1.4)
Any unsolicited AEs of grade 3	4 (1.4)	0
Unsolicited AEs thought to be related to study vaccine	30 (10.8)	7 (5.1)
SAE^b^	9 (3.2)	6 (4.3)
SAEs thought to be related to study vaccine	0	0

^a^AEs by preferred term are those occurring in at least 3 participants overall.

^b^In the ExPEC10V group, 9 participants reported the following SAEs: abdominal hernia, infectious enterocolitis (reported post dose), pyelonephritis, sepsis, osteoarthritis, urinary calculus, and nephrolithiasis, each in 1 participant; and non-cardiac chest pain in 2 participants. In the placebo group, 6 participants reported the following SAEs: acute pyelonephritis, urinary tract infection, intervertebral disc protrusion, migraine, and chronic kidney disease, each in 1 participant. In addition, enterococcal sepsis, lumbar vertebral fracture, and pulmonary embolism were all reported in the same participant in the placebo group.

Data presented are n (%) unless otherwise specified. Participants are counted only once for any given event, regardless of the number of times they experienced the event. There were no (solicited or unsolicited) AEs of grade 4. Solicited AEs continuing beyond Day 15 were reported as unsolicited AEs. Grade 3 AEs are defined as severe: Symptoms causing inability to perform usual social and functional activities and requires medical intervention. *AE* adverse event, *SAE* serious adverse event, *SD* standard deviation.

Approximately half (50.4% [*n* = 70/139]) of ExPEC10V participants and 9.1% (*n* = 2/22) of placebo participants who had solicited local AEs experienced them as late-onset (first onset after Day 5). Most ExPEC10V participants (86.7% [*n* = 52/60] and 86.4% [38/44]) who had erythema and swelling experienced them as late onset. In contrast, 39.6% (*n* = 55/139) of ExPEC10V participants and 20.8% (*n* = 11/53) of placebo participants who had solicited systemic AEs experienced them as late onset. The incidence of AEs over time is shown in Supplementary Fig. 1. For the most frequently reported solicited systemic AEs, there was an approximately equal distribution between early and late-onset AEs in ExPEC10V participants, with fatigue, headache, and myalgia reported as late onset in 51.0%, 47.3%, and 51.2% of ExPEC10V participants who experienced them.

Comparable rates of unsolicited AEs were observed with ExPEC10V (28.4%) and placebo (26.1%) (Table 2). The most frequently reported unsolicited AEs were UTI (ExPEC10V, 2.5%; placebo, 7.2%), myalgia (ExPEC10V, 2.5%; placebo, 4.3%), and injection-site pruritis (ExPEC10V, 2.5%; placebo, 0%). Unsolicited AEs of grade 3 were reported by 1.4% (*n* = 4) of ExPEC10V participants and no placebo participants. One grade 3 event of vaccination-site pain and 1 grade 3 event of increased systolic blood pressure were considered related to the study vaccine. The other 2 unsolicited AEs of grade 3, fatigue and infectious enterocolitis, were considered not related to the study vaccine. Fifteen SAEs were reported. One ExPEC10V participant (0.4%) reported an SAE in the 29-day postvaccination period (infectious enterocolitis; also reported above as unsolicited AE of grade 3). In the follow-up period, 9 SAEs were reported in ExPEC10V participants (3.2%) (abdominal hernia, infectious enterocolitis, pyelonephritis, sepsis, osteoarthritis, urinary calculus, and nephrolithiasis, each in 1 participant; and non-cardiac chest pain in 2 participants) and 6 in placebo participants (4.3%) (acute pyelonephritis, UTI, intervertebral disc protrusion, migraine, and chronic kidney disease, each in 1 participant; and enterococcal sepsis, lumbar vertebral fracture, and pulmonary embolism were reported in 1 participant). None of the SAEs were considered related to the vaccine, and no deaths were reported. There were no discontinuations due to AEs or SAEs. Furthermore, there were no clinically meaningful findings in vital sign measurements, physical examination assessments, or other observations related to safety in this study.

### Immunogenicity

**Fig. 3:**
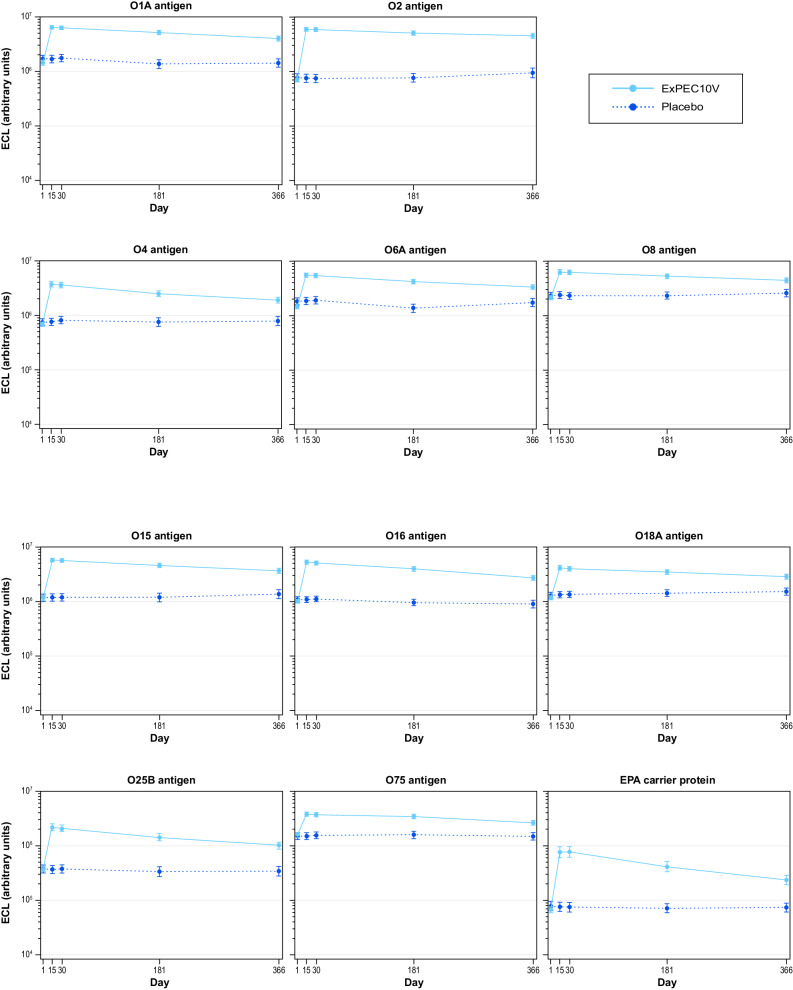
Multiplex ECL-determined IgG geometric mean titers (95% CI). ECL electrochemiluminescent-based immunoassay, EPA a genetically detoxified form of exotoxin A derived from *Pseudomonas aeruginosa*, IgG immunoglobulin G.

ECL results demonstrated that ExPEC10V elicited a robust immunogenic IgG antibody response across all tested vaccine serotypes (Fig. 3, Supplementary Table 2). The geometric mean fold increase from baseline to Day 15 by ECL ranged from 2.42 to 7.96 across serotypes, with 52.6% to 88.9% of participants exhibiting at least a 2-fold increase from baseline across serotypes. The geometric mean fold increase from baseline to Day 30 ranged from 2.33 to 8.18 across serotypes. At least a 2-fold increase in the Day 30 antibody response by ECL was observed in 51.2–89.1% of ExPEC10V-treated participants across serotypes. The percentage of participants exhibiting a 4-fold increase in antibody response on Day 30 ranged from 23.6% to 77.5% across serotypes. Minimal differences were observed across serotypes on the ECL between Days 15 and 30. On Day 181, antibody responses decreased across serotypes (geometric mean fold increase from baseline to Day 181, 2.14–6.79). A further decrease was observed on Day 366 (Year 1); however, antibody levels remained above placebo control levels for all serotypes with ECL (geometric mean fold increase from baseline, 1.68–6.05).

The percentage of participants having at least 7 serotypes with a 2-fold and 4-fold increase from baseline observed at Day 15, 30, 181, and 366 by ECL was 73.9% and 38.7%, 72.8% and 36.4%, 61.8% and 18.8%, and 46.2% and 7.1%, respectively (Supplementary Table 4).

**Fig. 4:**
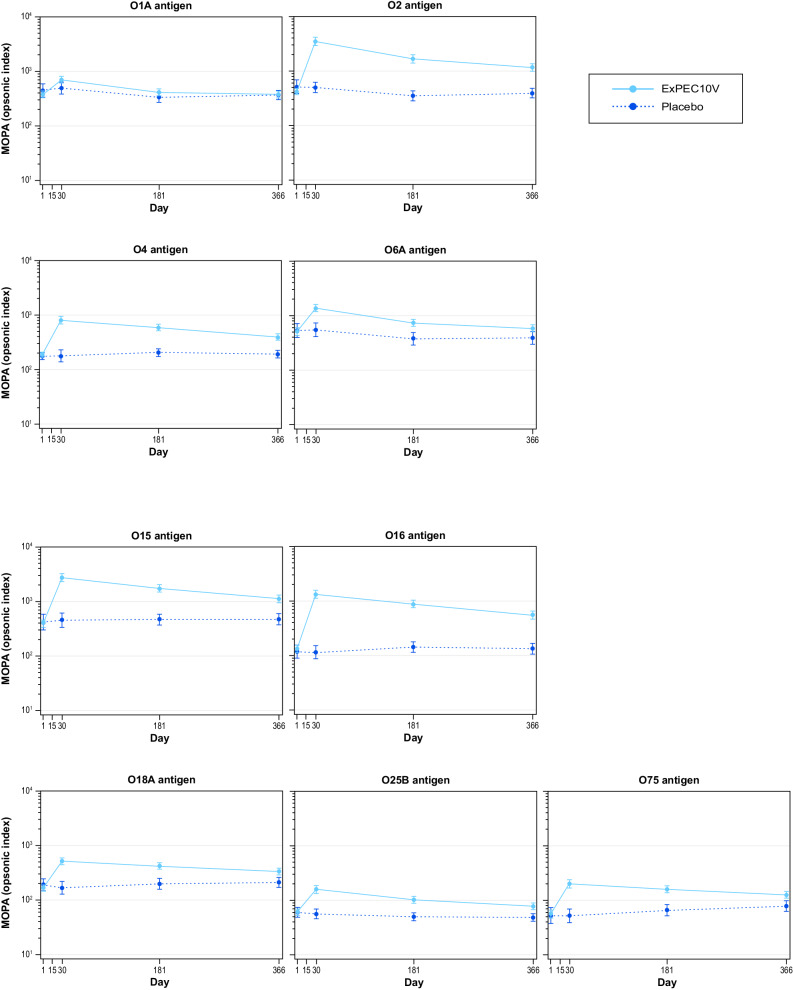
MOPA-determined functional antibody geometric mean titers (95% CI). IgG immunoglobulin G, MOPA multiplex opsonophagocytic assay.

Functional opsonophagocytic killing of *E. coli* strains was observed by MOPA following ExPEC10V across 9 of the 10 vaccine serotypes (O8 was not measured based on Cohort 1 results; Fig. 4 and Supplementary Table 3). MOPA geometric mean fold increases from baseline to Day 30 ranged from 1.81 to 9.68 across serotypes. All serotypes except O1A exhibited at least a 2-fold increase in GMT from baseline to Day 30. The percentage of ExPEC10V-treated participants exhibiting at least a 2-fold or 4-fold increase in antibody response by MOPA ranged from 41.1% to 86.0% and 17.8% to 70.9% across serotypes, respectively. Antibody responses decreased across serotypes on Day 181 (1.07–6.63) and on Day 366 (0.99–4.06).

The percentage of participants having at least 7 serotypes with a 2-fold and 4-fold increase from baseline observed at Day 30, 181, and 366 by MOPA was 39.9% and 13.6%, 22.0% and 2.7%, and 13.1% and 4.0%, respectively (Supplementary Table 4).

## Discussion

Previously published studies^24–27^, as well as data described here, demonstrate that vaccines targeting the ExPEC O-antigen, a component of the surface lipopolysaccharide, are safe and immunogenic in humans. Surface O-antigens are important virulence factors contributing to ExPEC pathogenicity^23,28^. Epidemiological data are key in the development of an effective prophylactic vaccine against IED^23,29,30^.

ExPEC10V and its precursor ExPEC4V were designed to target the most common causative ExPEC serotypes driving IED^23,27,29,30^. ExPEC4V, a 4-valent *E. coli* bioconjugate vaccine, targeted ExPEC O-antigens O1A, O2, O6A, and O25B and was immunogenic across the 4 serotypes in healthy adults^25,26,31^. In this trial, ExPEC10V demonstrated a robust functional antibody response for serotypes O1A, O2, O4, O6A, O15, O16, O18A, O25B, and O75. The geometric mean titers of serotype-specific antibodies, as measured by ECL, increased substantially by Day 15 and remained above the levels observed in the placebo group through Year 1. Similarly, functional antibody responses, as measured by MOPA, were observed on Day 30 and persisted above placebo control levels for all serotypes except O1A through Year 1. Decreases in functional antibody titers at 1 year were also reported for the 13-valent pneumococcal conjugate vaccine^32^ but did not appear to impact vaccine efficacy as no waning of efficacy was observed for the 5-year duration of the CAPiTA study^33^. The ongoing phase 3 ExPEC9V trial will further investigate durability of antibody responses.

The inclusion of participants with a history of UTI supports the plan for late-stage development of ExPEC10V with a focus on a high-risk population that can drive efficiency and cost reduction in phase 3 vaccine efficacy trials. The ongoing, pivotal phase 3 ExPEC9V vaccine trial, E.mbrace (VAC52416BAC3001, NCT04899336), is examining vaccine efficacy in participants ≥60 years with a history of UTI in the past 2 years.

Data presented here provide evidence supporting the acceptable safety profile and robust immunogenic response following ExPEC10V vaccination in elderly healthy adults with a history of UTI. Solicited AEs occurred in 64.4% of ExPEC10V participants and 42.8% of placebo participants, with the most common events being pain/tenderness, fatigue, headache, and myalgia. No safety concerns were identified, and no SAEs related to the study vaccine occurred. Notably, late-onset solicited local AEs were common among ExPEC10V participants (50.4%) but not among placebo participants (9.1%). More than 85% of participants who had erythema and swelling experienced them as late onset. To date, the cause of this observation remains unexplained. In this study, all late-onset solicited local AEs were grade 1 or grade 2. The median duration of late-onset solicited systemic AEs was 2 days for participants in the ExPEC10V low-dose group and the ExPEC10V medium-dose group, and 1 day for participants in the ExPEC10V high-dose group.

Previous studies have reported similar findings regarding late-onset solicited local AEs with other vaccines, including diphtheria, tetanus, and pertussis toxoid^34^. The cause of this is likely to be multifactorial, with age being a contributing factor, and warrants further investigation into the pathophysiology^34^. Importantly, this analysis examined the safety, reactogenicity, and immunogenicity of ExPEC10V specifically in participants with a documented history of UTI, an identified risk factor for IED^4^. The results from this Cohort 2 showed a comparable antibody response to the earlier Cohort 1 of this study with the exception of serotype O1A, which shows similar binding antibody titers but a lower functional antibody response in the current study^24^. A potential reason for this change could be due to different assay versions being used between cohorts, which is currently being investigated.

This study is limited in that results may not be generalizable in participants <60 years old or in geographical regions outside the study site locations. Further, safety and immunogenicity data beyond 1-year post vaccination are not yet available for ExPEC10V. Data beyond 1-year post vaccination will be generated in Cohort 1 wherein participants are followed until Year 5.

The intestinal colonization of various O serotypes in different populations and over time has not been carefully studied, however *E. coli* O serotypes linked to invasive disease or bacteremia have been shown to be stable over several decades^1,3^. Furthermore, designing vaccines against multiple pathovars has proved to be challenging^35^. The variability of *E. coli* virulence factors dictates that a vaccine against any target pathovar must be formulated of several components to provide additive protection or be composed of a multi-serotype formulation in case of a dominated targeted virulence factor^1^. However, even within pathovar boundaries, immunological heterogeneity proves to be challenging for making multi-formulated vaccines for broad protection. For example, some heat-stable enterotoxins can remain resistant to vaccine development, even though they are conserved antigens^36^. There is a strong unmet need for preventive measures against IED, particularly given the context of growing antimicrobial-resistant strains of *E. coli* across regions^6,7,9,10^. Collectively, these data contribute to the acceptable safety profile and strong immunogenicity response profile observed with ExPEC10V in participants with a history of UTI. Of note, the MOPA immunogenicity analysis of Cohort 1 showed that the O8 strain used in the assay for clinical testing was not able to discriminate a vaccine-induced immune response at Day 15 linked to high baseline titers, necessitating additional assay optimization. Therefore, a reformulated 9-valent serotype vaccine, ExPEC9V, excluding O8, was advanced for further clinical development in a phase 3 study (E.mbrace, NCT04899336) that may provide valuable data regarding the efficacy of ExPEC9V for the prevention of IED.

## Methods

### Study design and participants

This was a randomized, multicenter, interventional, phase 1/2a study conducted across 27 sites in the United States and Europe (https://clinicaltrials.gov/study/NCT03819049). The study was reviewed and approved by the Institutional Review Board and/or independent ethics committee of each study site before the start of the study. All procedures were conducted in accordance with the ethical principles of the Declaration of Helsinki and with Good Clinical Practice. All participants provided written informed consent.

The study was initiated on 6 July 2020 (the date of enrollment of the first participant in Cohort 2), and the study was completed on 6 April 2022 (date of last contact with the last participant). Cohort 2 enrollment was initiated after the primary analysis of Cohort 1^24^. The study utilized a double-blind, placebo-controlled design and comprised a maximum 28-day screening, vaccination (Day 1), double-blind 181-day follow-up, and long-term follow-up until Year 1 (end of the study for Cohort 2). Healthy adults ≥60 years of age with a documented history of UTI within the past 5 years were enrolled. Participants were required to have a body mass index of >18.5 to <40 kg/m^2^. Individuals were excluded from the study if they had an acute illness, with ongoing or suspected UTI, or a history of an underlying clinically significant (acute or uncontrolled chronic) medical condition for which the investigator deemed participation in the study was not in their best interest. Female participants were required to be postmenopausal and not intending to conceive by any method. Full inclusion and exclusion criteria are listed in the supplemental material (Supplementary Table 1).

Participants were randomized 2:1 to receive ExPEC10V (Table 3) or placebo. All participants were centrally assigned to randomized study vaccination with an interactive web response system based on a computer-generated randomization schedule prepared before the study by or under the supervision of the sponsor. Participants, clinical staff, investigators, and sponsor personnel, except for the designated pharmacist or qualified staff member with the primary responsibility of preparing and administering the study vaccine, were blinded to study vaccination group allocation. The study vaccine was administered by a blinded vaccine administrator, study nurse, medical doctor, or otherwise qualified healthcare professional. Venous blood samples for immunogenicity analyses were collected at baseline (Day 1/pre vaccination) and on Days 15, 30, 181, and 366 (Year 1). Concomitant therapies were permitted and required to be recorded for all participants from 30 days prior to vaccination until 30 days after vaccination.

**Table 3:** Study vaccination

Study vaccination	O1A (µg)	O2 (µg)	O4 (µg)	O6A (µg)	O8 (µg)	O15 (µg)	O16 (µg)	O18A (µg)	O25B (µg)	O75 (µg)	EPA (µg)	PS (Tot) (µg)
**ExPEC10V**	8	8	8	8	8	8	8	8	16	8	319	88

ExPEC10V consisted of the O-antigen PSs of the ExPEC serotypes O1A, O2, O4, O6A, O8, O15, O16, O18A, O25B, and O75 separately bioconjugated to the EPA carrier protein. The EPA (μg) was calculated using a ratio of 0.276 for PS/EPA. However, the final EPA dose was confirmed at the release. EPA, a genetically detoxified form of exotoxin A derived from *Pseudomonas aeruginosa*; *PS* polysaccharide, *Tot* total.

### Study drug

All participants were administered a single 0.5-mL intramuscular injection of ExPEC10V (Table 3) or placebo (normal saline) to the deltoid muscle. ExPEC10V is an *E. coli* bioconjugate vaccine in phosphate-buffered solution containing O-antigen polysaccharides of ExPEC serotypes O1A, O2, O4, O6A, O8, O15, O16, O18A, O25B, and O75 separately bioconjugated to a genetically detoxified form of the carrier protein exotoxin A derived from *Pseudomonas aeruginosa* (EPA).

### Assessments

#### Safety and reactogenicity

Primary endpoints assessed were solicited local and systemic adverse events (AEs) collected until Day 15 (14 days post vaccination), unsolicited AEs collected until Day 30 (29 days post vaccination), and serious AEs (SAEs) collected until Day 181 (180 days post vaccination). Solicited AEs continuing after Day 15 were recorded as unsolicited AEs. SAEs related to the study vaccine or study procedures collected from Day 182 until the end of the study (Year 1) were assessed as a secondary endpoint. Any clinically meaningful changes observed on physical examinations, vital sign measurements, and clinical laboratory tests were recorded as AEs.

#### Immunogenicity

Vaccine serotype-specific antibody titers elicited by the vaccine, as measured on Day 30 via electrochemiluminescent-based immunoassay (ECL) and multiplex opsonophagocytic assay (MOPA), were assessed as primary endpoints. A qualified multiplex ECL was used to determine the levels of immunoglobulin G (IgG) antibodies against *E. coli* vaccine serotypes and the carrier protein EPA, and a validated MOPA assay was used as a functional assay evaluating the ability of antibodies to mediate opsonophagocytic killing of *E. coli* vaccine serotypes. Immunogenicity assays were performed according to previously published methods^24^. The analysis of Cohort 1^24^ showed that the MOPA O8 strain used was not able to discriminate a vaccine-induced response at baseline and on Day 15. As the sponsor decided to progress with clinical development with a new formulation (ExPEC9V) of the vaccine excluding serotype O8, the MOPA was not performed for this serotype for Cohort 2. Assessments on Days 15 and 181 and Year 1 for the ECL and assessments on Day 181 and Year 1 for the MOPA were secondary endpoints.

#### Statistical analysis

The full analysis set (FAS) included all randomized participants with a vaccine administration documented. Safety data were evaluated using the FAS. The per protocol immunogenicity (PPI) analysis set included all randomized and vaccinated participants for whom immunogenicity data were available, excluding those samples with major protocol deviations expected to impact the immunogenicity outcomes. Immunogenicity data were evaluated using the PPI analysis set. An ExPEC10V group sample size of 280 participants was estimated to provide 95% confidence that if no AE or SAE is observed, the true incidence is ≤1.1%. Descriptive statistics were used to assess safety and immunogenicity data. For immunogenicity endpoints, antibody geometric mean titer (GMT), geometric mean fold increase from baseline, and the percentage of participants exhibiting at least a 2-fold or 4-fold increase in antibody response were used to evaluate differences observed between groups.

### Reporting summary

Further information on research design is available in the Nature Research Reporting Summary linked to this article.

### Supplementary information

Supplemental material

REPORTING SUMMARY

## Data Availability

Although these data are not currently publicly available for sharing, requests for sharing can be sent to the Corresponding Author and will be evaluated on an individual basis.
