# Supplementary material for: A randomized phase 1/2a trial of ExPEC10V vaccine in adults with a history of UTI
Source: NPJ Vaccines. 2024 Jun 14;9:106. doi: 10.1038/s41541-024-00885-1 (PMC11178786; doi:10.1038/s41541-024-00885-1)
Supplement: Supplementary file 1 — Supplemental material [file 41541_2024_885_MOESM1_ESM.pdf]

Supplemental Table 1. Inclusion and exclusion criteria.

**Inclusion Criteria**
Male or female, ≥60 years of age for Cohort 2, inclusive, on the day of signing the informed consent form (ICF) and available for the duration of the study
Must have a body mass index of >18.5 to <40 kg/m^2
Must be healthy or medically stable, in the investigator's clinical judgment, as confirmed by medical history, physical examination and vital signs, performed at the screening visit. Participan**ts** may have underlying illnesses, such as hypertension, diabetes, or ischemic heart disease, as long as their symptoms/signs are medically controlled. If the participant is on medication for a condition, the medication dose must have been stable for at least 12 weeks preceding vaccination and expected to remain stable for the duration of the study (or minor dose adaptations could be done and accepted based on physician's judgment)
Before randomization, a woman must be:<br>a. Postmenopausal (a postmenopausal state is defined as no menses for 12 months without an alternative medical cause); and<br>b. Not intending to conceive by any methods
Must sign an ICF indicating that he or she understands the purpose of, and procedures required for, the study and is willing to participate in the study
Must be willing and able to adhere to the lifestyle restrictions specified in this protocol
Agrees not to donate blood until 12 weeks after receiving the study vaccine
Must be willing to provide verifiable identification, has means to be contacted and to contact the investigator during the study
Must have a documented history of urinary tract infection in the past 5 years
**Exclusion Criteria**
Acute illness (this does not include minor illnesses such as diarrhea or mild upper respiratory tract infection) or temperature ≥38.0°C (100.4°F) within 24 hours prior to the administration of study vaccine. Enrollment at a later date within the screening window of 28 days was permitted.
History of malignancy within 5 years before screening (exceptions are squamous and basal cell carcinomas of the skin, carcinoma in situ of the cervix, or malignancy considered cured with minimal risk of recurrence)
Known allergies, hypersensitivity, or intolerance to ExPEC10V or its excipients
Contraindication to intramuscular injections and blood draws (eg, bleeding disorder)

Abnormal function of the immune system resulting from:<br>a. Clinical conditions (eg, autoimmune disease or immunodeficiency)<br>b. Chronic or recurrent use of systemic corticosteroids<br>*Note: Ocular, topical, or inhaled steroids are allowed*<br>c. Administration of antineoplastic and immunomodulating agents or radiotherapy
History of acute polyneuropathy (eg, Guillain-Barré syndrome)
History of chronic urticaria (recurrent hives), eczema, or atopic dermatitis
Received treatment with immunoglobulin therapy in the 2 months, or blood products in the 4 months, before the planned administration of the study vaccine or has any plans to receive such treatment during the study
Received or plans to receive:<br>a. Licensed live attenuated vaccines – within 28 days before or after planned administration of the study vaccine<br>b. Other licensed (not live) vaccines – within 14 days before or after planned administration of the study vaccine
Received an investigational drug or used an invasive investigational medical device or received an investigational vaccine within 90 days before the planned administration of the study vaccine, or is currently enrolled or plans to participate in another investigational study until Day 181 in this study<br>*Note: Participation in an observational clinical study is allowed with prior approval of the sponsor. During the long-term follow-up periods, participation in another investigational study is allowed only with prior approval of the sponsor*
History of an underlying clinically significant acute or uncontrolled chronic medical condition or physical examination findings for which, in the opinion of the investigator, participation would not be in the best interest of the participant (eg, compromise the well-being) or that could prevent, limit, or confound the protocol-specified assessments
Had major surgery (per the investigator's judgment) within 4 weeks prior to randomization, or has surgery planned during the time the participant is expected to participate in the study or within 6 months after the last study vaccine administration<br>*Note: Participants with planned surgical procedures to be conducted under local anesthesia and not judged as major by the investigator may participate*
Employee of the investigator or study site, with direct involvement in the proposed study or other studies under the direction of that investigator or study site, as well as family members of the employees or the investigator, or an employee of the sponsor
Evidence of chronic active hepatitis B or hepatitis C infection by medical history
Evidence of HIV type 1 or type 2 infection by medical history
Cannot communicate reliably with the investigator
Who, in the opinion of the investigator, is unlikely to adhere to the requirements of the study or is unlikely to complete the vaccination and subsequent follow-up

Who has had major psychiatric illness and/or drug substance or alcohol abuse in the past 12 months which in the investigator's opinion would compromise the participant's safety or compliance with the study procedure

Supplemental Table 2. Multiplex ECL-determined immunoassay IgG geometric mean titers, geometric mean fold increases, and least fold increases from baseline (Day 1) to Day 366 (Year 1).

Serotype/ Day	ECL: ExPEC10V				ECL: Placebo			
N=388	GMT (95% CI)	GM FI (95%CI)	% 2-fold[2]	% 4-fold[2]	GMT (95% CI)	GM FI (95%CI)	% 2-fold[2]	% 4-fold[2]
**O1A**								
**Day 1**	1452159.5 (1298653.3; 1623810.8)	–	–	–	1702503.6 (1452301.9; 1995809.8)	–	–	–
**Day 15**	6440111.0 (5964513.8; 6953631.4)	4.40 (3.932; 4.928)	76.3% (70.55%; 81.39%)	54.5% (48.19%; 60.79%)	1683620.1 (1434083.1; 1976577.8)	0.99 (0.967; 1.023)	0.0% (0.00%; 2.78%)	0.0% (0.00%; 2.78%)
**Day 30**	6341302.4 (5889782.4; 6827436.7)	4.40 (3.958; 4.893)	78.3% (72.76%; 83.17%)	54.7% (48.36%; 60.84%)	1762724.3 (1505477.9; 2063927.4)	1.02 (0.955; 1.095)	1.6% (0.19%; 5.49%)	0.8% (0.02%; 4.24%)
**Day 181**	5181579.3 (4717321.1; 5691527.8)	3.54 (3.186; 3.937)	71.4% (65.40%; 76.84%)	48.2% (41.96%; 54.55%)	1363698.1 (1134292.4; 1639500.3)	0.85 (0.778; 0.930)	2.5% (0.51%; 7.02%)	0.0% (0.00%; 2.98%)
**Day 366**	4022674.3 (3621999.8; 4467672.3)	2.77 (2.497; 3.070)	62.4% (56.09%; 68.32%)	34.9% (29.06%; 41.10%)	1425709.7 (1193504.1; 1703092.6)	0.83 (0.758; 0.909)	7.0% (3.24%; 12.83%)	0.0% (0.00%; 2.82%)
**O2**								
**Day 1**	734917.5 (655648.9; 823769.9)	–	–	–	770477.4 (649332.9; 914223.6)	–	–	–
**Day 15**	5984689.5 (5506341.8; 6504592.4)	7.96 (7.052; 8.987)	88.9% (84.40%; 92.52%)	77.9% (72.24%; 82.83%)	758680.9 (637710.3; 902599.0)	0.99 (0.933; 1.057)	0.8% (0.02%; 4.18%)	0.8% (0.02%; 4.18%)
**Day 30**	5957433.5 (5487954.2; 6467075.6)	8.18 (7.255; 9.227)	89.1% (84.70%; 92.67%)	77.5% (71.93%; 82.46%)	749984.6 (628913.9; 894362.5)	0.98 (0.909; 1.055)	1.6% (0.19%; 5.49%)	1.6% (0.19%; 5.49%)
**Day 181**	5104290.1 (4630408.3; 5626669.7)	6.79 (6.020; 7.649)	86.7% (81.94%; 90.62%)	69.9% (63.90%; 75.47%)	772833.5 (647611.4; 922268.6)	1.10 (0.964; 1.252)	12.2% (6.99%; 19.32%)	7.3% (3.40%; 13.44%)
**Day 366**	4539197.6 (4061044.2; 5073649.6)	6.05 (5.330; 6.878)	81.6% (76.26%; 86.13%)	66.3% (60.11%; 72.05%)	945957.9 (770610.6; 1161204.5)	1.21 (1.087; 1.342)	18.6% (12.30%; 26.41%)	3.9% (1.27%; 8.81%)
**O4**								
**Day 1**	674212.0 (612696.0; 741904.3)	–	–	–	742523.0 (644020.1; 856092.0)	–	–	–
**Day 15**	3674695.0 (3268908.7; 4130853.6)	5.45 (4.825; 6.146)	81.8% (76.50%; 86.37%)	60.9% (54.56%; 66.92%)	752878.3 (648044.1; 874671.5)	1.02 (0.988; 1.063)	1.5% (0.19%; 5.41%)	0.8% (0.02%; 4.18%)
**Day 30**	3537556.6 (3153145.2; 3968833.1)	5.31 (4.726; 5.955)	81.4% (76.10%; 85.95%)	58.9% (52.64%; 64.98%)	805295.5 (689894.4; 940000.1)	1.09 (1.014; 1.168)	2.3% (0.48%; 6.65%)	1.6% (0.19%; 5.49%)
**Day 181**	2464185.1 (2180166.2; 2785204.2)	3.68 (3.284; 4.116)	69.0% (62.95%; 74.64%)	43.5% (37.35%; 49.86%)	748466.8 (626770.4; 893792.2)	1.02 (0.948; 1.105)	3.3% (0.89%; 8.12%)	2.4% (0.51%; 6.96%)
**Day 366**	1877926.0 (1662018.9; 2121880.9)	2.82 (2.536; 3.144)	60.0% (53.70%; 66.06%)	32.5% (26.84%; 38.67%)	781470.1 (645829.6; 945598.4)	1.04 (0.951; 1.139)	11.6% (6.66%; 18.45%)	3.1% (0.85%; 7.75%)

**O6A**								
**Day 1**	1477311.2 (1340402.2; 1628204.1)	–	–	–	1842541.7 (1586132.6; 2140401.1)	–	–	–
**Day 15**	5549559.4 (5061238.2; 6084995.1)	3.89 (3.518; 4.298)	73.9% (68.04%; 79.21%)	49.8% (43.48%; 56.13%)	1864516.2 (1602306.3; 2169635.5)	1.03 (0.989; 1.070)	0.8% (0.02%; 4.18%)	0.0% (0.00%; 2.78%)
**Day 30**	5461039.4 (4992165.2; 5973951.2)	3.64 (3.289; 4.019)	73.3% (67.41%; 78.56%)	44.2% (38.03%; 50.48%)	1944280.6 (1664293.0; 2271371.1)	1.06 (0.993; 1.138)	2.3% (0.48%; 6.65%)	0.8% (0.02%; 4.24%)
**Day 181**	4237338.6 (3839748.2; 4676097.9)	2.88 (2.569; 3.222)	64.3% (58.10%; 70.19%)	39.2% (33.18%; 45.50%)	1377697.3 (1156472.5; 1641240.8)	0.78 (0.678; 0.894)	6.5% (2.85%; 12.41%)	0.8% (0.02%; 4.45%)
**Day 366**	3366305.0 (3030852.9; 3738884.7)	2.31 (2.102; 2.534)	54.7% (48.38%; 60.96%)	22.0% (17.11%; 27.65%)	1746191.5 (1476781.0; 2064750.9)	0.95 (0.875; 1.028)	2.3% (0.48%; 6.65%)	0.8% (0.02%; 4.24%)
**O8**								
**Day 1**	2159027.3 (1951448.0; 2388687.2)	–	–	–	2348258.5 (2048409.1; 2692000.2)	–	–	–
**Day 15**	6259628.9 (5787049.2; 6770800.1)	2.89 (2.619; 3.194)	62.1% (55.77%; 68.06%)	36.4% (30.43%; 42.62%)	2402212.7 (2080311.6; 2773923.8)	1.03 (0.992; 1.072)	1.5% (0.19%; 5.41%)	1.5% (0.19%; 5.41%)
**Day 30**	6240213.6 (5782238.7; 6734461.7)	2.92 (2.655; 3.221)	64.0% (57.77%; 69.82%)	38.4% (32.41%; 44.61%)	2315932.9 (2011986.2; 2665796.4)	1.01 (0.965; 1.058)	1.6% (0.19%; 5.49%)	1.6% (0.19%; 5.49%)
**Day 181**	5341568.9 (4901755.3; 5820845.0)	2.45 (2.239; 2.681)	55.9% (49.54%; 62.04%)	27.3% (21.98%; 33.24%)	2347175.9 (2014621.2; 2734625.6)	1.06 (1.000; 1.130)	3.3% (0.89%; 8.12%)	0.8% (0.02%; 4.45%)
**Day 366**	4468182.7 (4073515.2; 4901088.0)	2.09 (1.915; 2.277)	51.0% (44.67%; 57.27%)	18.4% (13.87%; 23.74%)	2612423.8 (2233155.6; 3056105.0)	1.10 (1.009; 1.202)	10.1% (5.48%; 16.62%)	3.1% (0.85%; 7.75%)
**O15**								
**Day 1**	1169835.3 (1049055.8; 1304520.3)	–	–	–	1190000.8 (1015187.8; 1394916.2)	–	–	–
**Day 15**	5820591.1 (5382542.2; 6294289.8)	4.93 (4.420; 5.505)	82.2% (76.93%; 86.72%)	58.9% (52.56%; 65.02%)	1196527.5 (1020092.0; 1403479.3)	1.02 (0.976; 1.070)	0.8% (0.02%; 4.18%)	0.8% (0.02%; 4.18%)
**Day 30**	5724175.1 (5301709.2; 6180305.1)	5.00 (4.483; 5.565)	84.1% (79.07%; 88.35%)	58.5% (52.25%; 64.60%)	1207724.5 (1028556.8; 1418101.9)	1.02 (0.961; 1.077)	1.6% (0.19%; 5.49%)	0.8% (0.02%; 4.24%)
**Day 181**	4651707.1 (4246266.4; 5095859.9)	4.08 (3.692; 4.514)	79.6% (74.13%; 84.38%)	52.5% (46.23%; 58.81%)	1212443.2 (1022590.8; 1437543.2)	1.08 (1.016; 1.150)	1.6% (0.20%; 5.75%)	0.8% (0.02%; 4.45%)
**Day 366**	3690523.9 (3327526.1; 4093120.9)	3.15 (2.862; 3.468)	67.8% (61.73%; 73.53%)	36.9% (30.93%; 43.11%)	1379883.1 (1146231.7; 1661162.7)	1.18 (1.064; 1.312)	13.2% (7.87%; 20.26%)	7.0% (3.24%; 12.83%)
**O16**								
**Day 1**	1028916.1 (946185.6; 1118880.3)	–	–	–	1095597.3 (969037.9; 1238685.7)	–	–	–
**Day 15**	5328787.2 (4876101.2; 5823499.4)	5.14 (4.615; 5.715)	86.2% (81.29%; 90.17%)	62.5% (56.17%; 68.44%)	1094722.5 (967329.6; 1238892.4)	1.03 (0.984; 1.078)	0.8% (0.02%; 4.18%)	0.8% (0.02%; 4.18%)
**Day 30**	5158063.5 (4735530.7; 5618297.2)	5.04 (4.571; 5.561)	86.0% (81.21%; 90.03%)	62.4% (56.18%; 68.33%)	1127584.4 (1001022.6; 1270147.7)	1.06 (0.993; 1.126)	1.6% (0.19%; 5.49%)	1.6% (0.19%; 5.49%)

**Day 181**	4002647.6 (3633889.3; 4408826.6)	3.98 (3.605; 4.385)	78.8% (73.29%; 83.67%)	49.8% (43.50%; 56.11%)	974253.6 (851675.7; 1114473.6)	0.92 (0.859; 0.982)	0.0% (0.00%; 2.95%)	0.0% (0.00%; 2.95%)
**Day 366**	2732322.4 (2460092.5; 3034676.8)	2.63 (2.388; 2.906)	63.9% (57.70%; 69.82%)	27.5% (22.07%; 33.37%)	909470.4 (771204.3; 1072525.6)	0.83 (0.753; 0.920)	2.3% (0.48%; 6.65%)	0.8% (0.02%; 4.24%)
**O18A**								
**Day 1**	1193010.6 (1089212.5; 1306700.2)	–	–	–	1307064.2 (1146644.3; 1489927.4)	–	–	–
**Day 15**	4224096.2 (3810089.9; 4683088.6)	3.63 (3.266; 4.031)	69.2% (63.08%; 74.80%)	47.4% (41.14%; 53.78%)	1343124.1 (1178653.4; 1530545.3)	1.06 (1.023; 1.093)	0.8% (0.02%; 4.18%)	0.0% (0.00%; 2.78%)
**Day 30**	4046778.4 (3656809.6; 4478334.1)	3.40 (3.088; 3.754)	69.0% (62.96%; 74.58%)	43.0% (36.90%; 49.31%)	1365154.9 (1196273.0; 1557878.4)	1.07 (1.017; 1.132)	1.6% (0.19%; 5.49%)	0.8% (0.02%; 4.24%)
**Day 181**	3510981.8 (3167417.1; 3891812.4)	2.91 (2.657; 3.179)	62.4% (56.09%; 68.32%)	34.9% (29.06%; 41.10%)	1440992.4 (1251837.9; 1658728.5)	1.16 (1.088; 1.234)	2.4% (0.51%; 6.96%)	0.0% (0.00%; 2.95%)
**Day 366**	2902883.8 (2610124.6; 3228479.7)	2.44 (2.248; 2.658)	55.3% (48.96%; 61.50%)	24.3% (19.18%; 30.06%)	1538291.3 (1327257.2; 1782880.0)	1.19 (1.106; 1.286)	11.6% (6.66%; 18.45%)	0.0% (0.00%; 2.82%)
**O25B**								
**Day 1**	381947.7 (336249.7; 433856.4)	–	–	–	377595.0 (318648.3; 447446.3)	–	–	–
**Day 15**	2205580.7 (1907527.9; 2550204.4)	5.80 (4.990; 6.748)	77.1% (71.40%; 82.11%)	58.1% (51.76%; 64.25%)	369020.0 (309515.7; 439964.0)	1.01 (0.963; 1.068)	0.8% (0.02%; 4.18%)	0.8% (0.02%; 4.18%)
**Day 30**	2117662.8 (1843164.1; 2433042.1)	5.59 (4.866; 6.428)	78.3% (72.76%; 83.17%)	56.6% (50.30%; 62.72%)	375570.5 (316412.1; 445789.5)	1.04 (0.963; 1.115)	2.3% (0.48%; 6.65%)	1.6% (0.19%; 5.49%)
**Day 181**	1448059.1 (1242167.0; 1688078.3)	3.73 (3.237; 4.300)	65.5% (59.30%; 71.31%)	44.3% (38.12%; 50.64%)	338325.5 (276179.7; 414455.2)	0.95 (0.814; 1.102)	10.6% (5.75%; 17.40%)	6.5% (2.85%; 12.41%)
**Day 366**	1010150.2 (874663.8; 1166623.5)	2.66 (2.365; 2.983)	56.9% (50.54%; 63.03%)	30.6% (24.99%; 36.64%)	344740.3 (281210.9; 422621.9)	0.91 (0.830; 1.003)	6.2% (2.72%; 11.85%)	2.3% (0.48%; 6.65%)
**O75**								
**Day 1**	1607834.9 (1462172.3; 1768008.6)	–	–	–	1526460.5 (1325349.6; 1758088.4)	–	–	–
**Day 15**	3879088.3 (3534225.0; 4257602.7)	2.42 (2.217; 2.644)	52.6% (46.22%; 58.86%)	24.5% (19.34%; 30.28%)	1525428.5 (1313220.7; 1771927.8)	1.02 (0.977; 1.071)	1.5% (0.19%; 5.41%)	1.5% (0.19%; 5.41%)
**Day 30**	3758021.3 (3419541.0; 4130005.8)	2.33 (2.136; 2.531)	51.2% (44.89%; 57.41%)	23.6% (18.59%; 29.31%)	1577900.4 (1365705.2; 1823065.1)	1.04 (0.979; 1.110)	2.3% (0.48%; 6.65%)	2.3% (0.48%; 6.65%)
**Day 181**	3483252.0 (3157048.6; 3843160.4)	2.14 (1.983; 2.307)	50.8% (44.48%; 57.06%)	16.0% (11.74%; 21.09%)	1605445.2 (1367665.3; 1884565.2)	1.11 (1.029; 1.196)	4.9% (1.81%; 10.32%)	3.3% (0.89%; 8.12%)
**Day 366**	2675947.8 (2416208.1; 2963609.2)	1.68 (1.563; 1.809)	33.7% (27.95%; 39.89%)	8.2% (5.17%; 12.31%)	1512970.1 (1293159.3; 1770144.2)	1.00 (0.917; 1.088)	8.6% (4.37%; 14.86%)	0.8% (0.02%; 4.28%)

Data presented are from the per protocol immunogenicity analysis set. 95% CI for GMT and GM FI is based on the t-distribution. ECL, electrochemiluminescent-based assay; GM FI, geometric mean fold increase from baseline; GMT, geometric mean titer; IgG, immunoglobulin G; LLOQ, lower limit of quantification.

Supplemental Table 3. MOPA geometric mean titers, geometric mean fold increases, and least fold increases from baseline (Day 1) to Day 366 (Year 1).

**Serotype/ Day**	**MOPA: ExPEC10V**				**MOPA: Placebo**			
N=388	**GMT (95% CI)**	**GM FI (95%CI)**	**% 2-fold[2]**	**% 4-fold[2]**	**GMT (95% CI)**	**GM FI (95%CI)**	**% 2-fold[2]**	**% 4-fold[2]**
**O1A**								
**Day 1**	377.8 (327.5; 435.8)	–	–	–	443.7 (333.7; 589.9)	–	–	–
**Day 15**	–	–	–	–	–	–	–	–
**Day 30**	703.0 (611.1; 808.7)	1.81 (1.602; 2.054)	41.5% (35.40%; 47.75%)	17.8% (13.36%; 23.06%)	492.4 (382.9; 633.2)	1.23 (1.036; 1.451)	22.9% (13.67%; 34.45%)	2.9% (0.35%; 9.94%)
**Day 181**	411.3 (355.6; 475.7)	1.07 (0.948; 1.201)	22.1% (17.18%; 27.66%)	8.5% (5.42%; 12.63%)	334.1 (270.8; 412.3)	0.80 (0.671; 0.954)	12.5% (5.88%; 22.41%)	1.4% (0.04%; 7.50%)
**Day 366**	376.0 (325.3; 434.5)	0.99 (0.880; 1.104)	22.6% (17.60%; 28.29%)	5.6% (3.07%; 9.15%)	368.5 (302.4; 449.1)	0.84 (0.704; 1.013)	12.3% (5.80%; 22.12%)	2.7% (0.33%; 9.55%)
**O2**								
**Day 1**	424.0 (372.4; 482.7)	–	–	–	519.1 (390.2; 690.5)	–	–	–
**Day 15**	–	–	–	–	–	–	–	–
**Day 30**	3523.2 (2950.7; 4206.7)	8.04 (6.684; 9.681)	81.0% (75.68%; 85.61%)	63.6% (57.37%; 69.45%)	502.4 (403.6; 625.4)	1.07 (0.901; 1.265)	18.6% (10.28%; 29.66%)	4.3% (0.89%; 12.02%)
**Day 181**	1684.8 (1413.3; 2008.5)	3.94 (3.285; 4.727)	64.7% (58.56%; 70.55%)	42.6% (36.52%; 48.92%)	352.1 (284.7; 435.5)	0.80 (0.658; 0.983)	12.5% (5.88%; 22.41%)	2.8% (0.34%; 9.68%)
**Day 366**	1167.9 (994.6; 1371.5)	2.70 (2.292; 3.178)	54.0% (47.60%; 60.24%)	32.1% (26.42%; 38.29%)	395.5 (322.9; 484.5)	0.78 (0.653; 0.931)	11.0% (4.85%; 20.46%)	1.4% (0.03%; 7.40%)
**O4**								
**Day 1**	187.4 (167.8; 209.3)	–	–	–	175.5 (136.4; 226.0)	–	–	–
**Day 15**	–	–	–	–	–	–	–	–
**Day 30**	803.9 (683.3; 945.7)	4.26 (3.607; 5.033)	64.7% (58.56%; 70.55%)	45.7% (39.54%; 52.03%)	178.7 (138.7; 230.4)	0.98 (0.827; 1.162)	14.3% (7.07%; 24.71%)	4.3% (0.89%; 12.02%)
**Day 181**	591.2 (512.8; 681.6)	3.16 (2.716; 3.686)	61.6% (55.39%; 67.59%)	36.4% (30.55%; 42.63%)	204.3 (173.2; 241.0)	1.15 (0.988; 1.349)	19.4% (11.06%; 30.47%)	2.8% (0.34%; 9.68%)
**Day 366**	398.1 (347.2; 456.5)	2.14 (1.857; 2.474)	48.0% (41.70%; 54.37%)	23.0% (17.97%; 28.71%)	191.7 (163.3; 225.0)	1.04 (0.889; 1.222)	11.0% (4.85%; 20.46%)	2.7% (0.33%; 9.55%)
**O6A**								
**Day 1**	516.6 (437.0; 610.7)	–	–	–	539.3 (400.9; 725.5)	–	–	–
**Day 15**	–	–	–	–	–	–	–	–
**Day 30**	1392.6 (1196.9; 1620.3)	2.57 (2.251; 2.939)	54.3% (47.97%; 60.46%)	32.9% (27.24%; 39.05%)	557.0 (416.8; 744.3)	1.02 (0.870; 1.192)	17.1% (9.18%; 28.03%)	1.4% (0.04%; 7.70%)
**Day 181**	747.6 (643.4; 868.6)	1.41 (1.244; 1.599)	31.4% (25.78%; 37.44%)	12.0% (8.31%; 16.62%)	379.7 (291.9; 494.0)	0.80 (0.680; 0.938)	9.7% (4.00%; 19.01%)	1.4% (0.04%; 7.50%)

**Day 366**	592.2 (509.2; 688.9)	1.11 (0.987; 1.259)	25.1% (19.86%; 30.94%)	9.2% (5.90%; 13.43%)	395.4 (302.6; 516.7)	0.84 (0.693; 1.009)	12.3% (5.80%; 22.12%)	6.8% (2.26%; 15.26%)
**O15**								
**Day 1**	395.3 (340.0; 459.5)	–	–	–	425.5 (305.9; 591.9)	–	–	–
**Day 15**	–	–	–	–	–	–	–	–
**Day 30**	2773.8 (2347.4; 3277.6)	6.70 (5.641; 7.958)	81.4% (76.10%; 85.95%)	62.8% (56.58%; 68.71%)	458.0 (338.4; 619.9)	1.05 (0.807; 1.376)	22.9% (13.67%; 34.45%)	8.6% (3.21%; 17.73%)
**Day 181**	1756.2 (1502.7; 2052.5)	4.19 (3.568; 4.919)	71.7% (65.79%; 77.12%)	51.2% (44.89%; 57.41%)	469.6 (372.5; 592.2)	1.13 (0.885; 1.431)	23.6% (14.40%; 35.09%)	5.6% (1.53%; 13.62%)
**Day 366**	1125.8 (958.3; 1322.7)	2.66 (2.251; 3.137)	57.9% (51.58%; 64.10%)	37.3% (31.31%; 43.59%)	478.8 (377.2; 607.7)	0.98 (0.736; 1.314)	21.9% (13.08%; 33.14%)	6.8% (2.26%; 15.26%)
**O16**								
**Day 1**	133.1 (114.6; 154.6)	–	–	–	118.8 (90.4; 156.2)	–	–	–
**Day 15**	–	–	–	–	–	–	–	–
**Day 30**	1347.0 (1132.2; 1602.5)	9.68 (8.055; 11.639)	86.0% (81.21%; 90.03%)	70.9% (64.98%; 76.40%)	116.0 (88.0; 152.8)	1.02 (0.841; 1.227)	18.6% (10.28%; 29.66%)	4.3% (0.89%; 12.02%)
**Day 181**	890.9 (758.7; 1046.1)	6.63 (5.601; 7.844)	79.8% (74.42%; 84.57%)	63.2% (56.97%; 69.08%)	144.5 (115.9; 180.1)	1.21 (0.994; 1.483)	25.4% (15.77%; 37.08%)	7.0% (2.33%; 15.67%)
**Day 366**	557.6 (470.4; 660.9)	4.06 (3.448; 4.782)	67.5% (61.30%; 73.21%)	49.6% (43.27%; 55.95%)	134.5 (107.8; 167.8)	0.98 (0.813; 1.174)	21.9% (13.08%; 33.14%)	0.0% (0.00%; 4.93%)
**O18A**								
**Day 1**	166.2 (145.3; 190.2)	–	–	–	189.9 (147.8; 244.1)	–	–	–
**Day 15**	–	–	–	–	–	–	–	–
**Day 30**	516.3 (446.2; 597.3)	2.88 (2.482; 3.332)	55.8% (49.52%; 61.97%)	34.5% (28.71%; 40.64%)	168.2 (128.8; 219.6)	0.93 (0.805; 1.083)	14.3% (7.07%; 24.71%)	0.0% (0.00%; 5.13%)
**Day 181**	423.9 (370.3; 485.2)	2.30 (2.008; 2.641)	53.5% (47.20%; 59.70%)	22.9% (17.89%; 28.48%)	199.7 (158.7; 251.2)	1.03 (0.875; 1.223)	16.7% (8.92%; 27.30%)	2.8% (0.34%; 9.68%)
**Day 366**	335.1 (291.5; 385.1)	1.84 (1.612; 2.097)	38.5% (32.45%; 44.80%)	17.1% (12.63%; 22.29%)	211.6 (171.5; 261.1)	0.97 (0.831; 1.135)	9.9% (4.06%; 19.26%)	2.8% (0.34%; 9.81%)
**O25B**								
**Day 1**	60.9 (<LLOQ; 69.5)	–	–	–	60.5 (<LLOQ; 74.5)	–	–	–
**Day 15**	–	–	–	–	–	–	–	–
**Day 30**	159.8 (135.4; 188.5)	2.05 (1.813; 2.308)	41.1% (35.02%; 47.36%)	21.3% (16.48%; 26.83%)	<LLOQ (<LLOQ; 69.6)	0.94 (0.841; 1.045)	5.7% (1.58%; 13.99%)	0.0% (0.00%; 5.13%)
**Day 181**	103.1 (88.9; 119.6)	1.42 (1.277; 1.571)	26.0% (20.73%; 31.77%)	11.6% (7.98%; 16.18%)	<LLOQ (<LLOQ; 59.1)	0.94 (0.857; 1.038)	4.2% (0.87%; 11.70%)	1.4% (0.04%; 7.50%)
**Day 366**	78.0 (67.6; 90.0)	1.19 (1.088; 1.294)	15.9% (11.59%; 20.98%)	5.6% (3.07%; 9.15%)	<LLOQ (<LLOQ; <LLOQ)	0.93 (0.822; 1.060)	8.2% (3.08%; 17.04%)	2.7% (0.33%; 9.55%)
**O75**								
**Day 1**	56.2 (47.5; 66.5)	–	–	–	52.6 (37.6; 73.5)	–	–	–

**Day 15**	–	–	–	–	–	–	–	–
**Day 30**	201.0 (169.5; 238.3)	3.21 (2.744; 3.745)	58.4% (52.12%; 64.55%)	36.9% (30.93%; 43.11%)	52.2 (39.2; 69.6)	1.18 (0.973; 1.438)	23.5% (14.09%; 35.38%)	10.3% (4.24%; 20.07%)
**Day 181**	160.4 (138.8; 185.3)	2.56 (2.246; 2.920)	55.5% (49.15%; 61.66%)	32.8% (27.09%; 38.93%)	66.6 (52.9; 83.8)	1.18 (0.965; 1.442)	29.6% (19.33%; 41.59%)	5.6% (1.56%; 13.80%)
**Day 366**	126.5 (109.7; 145.9)	2.09 (1.837; 2.369)	51.6% (45.22%; 57.94%)	24.8% (19.57%; 30.63%)	78.6 (62.6; 98.7)	1.40 (1.137; 1.717)	40.0% (28.47%; 52.41%)	5.7% (1.58%; 13.99%)

Data presented are from the per protocol immunogenicity analysis set. 95% CI for GMT and GM FI is based on the t-distribution. MOPA was not performed on D15 samples. GM FI, geometric mean fold increase from baseline; GMT, geometric mean titer; IgG, immunoglobulin G; LLOQ, lower limit of quantification; MOPA, multiplex opsonophagocytic assay.

Supplemental Table 4. Proportion of participants with multiplex ECL-determined immunoassay and MOPA for a given number of serotypes with 2- and 4-fold increase from baseline

	ECL				MOPA		
	**Day 15**	**Day 30**	**Day 181**	**Day 366**	**Day 30**	**Day 181**	**Day 366**
**Number of serotypes**	**N = 253**	**N = 258**	**N = 256**	**N = 255**	**N = 258**	**N = 258**	**N = 252**
**At least 2-fold change from baseline**							
**0**	2 (0.8%)	1 (0.4%)	1 (0.4%)	5 (2.0%)	0	5 (1.9%)	17 (6.7%)
**1**	4 (1.6%)	2 (0.8%)	3 (1.2%)	6 (2.4%)	7 (2.7%)	13 (5.0%)	24 (9.5%)
**2**	5 (2.0%)	6 (2.3%)	8 (3.1%)	12 (4.7%)	15 (5.8%)	25 (9.7%)	41 (16.3%)
**3**	6 (2.4%)	5 (1.9%)	12 (4.7%)	25 (9.8%)	24 (9.3%)	36 (14.0%)	37 (14.7%)
**4**	9 (3.6%)	7 (2.7%)	18 (7.0%)	21 (8.2%)	31 (12.0%)	39 (15.1%)	32 (12.7%)
**5**	17 (6.7%)	15 (5.8%)	20 (7.8%)	30 (11.8%)	40 (15.5%)	47 (18.2%)	37 (14.7%)
**6**	23 (9.1%)	34 (13.2%)	36 (14.1%)	38 (14.9%)	38 (14.7%)	36 (14.0%)	31 (12.3%)
**7**	34 (13.4%)	32 (12.4%)	40 (15.6%)	54 (21.2%)	46 (17.8%)	38 (14.7%)	23 (9.1%)
**8**	47 (18.6%)	54 (20.9%)	54 (21.1%)	34 (13.3%)	42 (16.3%)	13 (5.0%)	8 (3.2%)
**9**	67 (26.5%)	65 (25.2%)	48 (18.8%)	20 (7.8%)	15 (5.8%)	6 (2.3%)	2 (0.8%)
**10**	39 (15.4%)	37 (14.3%)	16 (6.3%)	10 (3.9%)			
**At least 4-fold change from baseline**							
**0**	13 (5.1%)	12 (4.7%)	15 (5.9%)	28 (11.0%)	12 (4.7%)	26 (10.1%)	60 (23.8%)
**1**	23 (9.1%)	19 (7.4%)	18 (7.0%)	42 (16.5%)	28 (10.9%)	47 (18.2%)	48 (19.0%)
**2**	9 (3.6%)	20 (7.8%)	37 (14.5%)	48 (18.8%)	35 (13.6%)	57 (22.1%)	60 (23.8%)
**3**	20 (7.9%)	18 (7.0%)	28 (10.9%)	40 (15.7%)	50 (19.4%)	39 (15.1%)	32 (12.7%)
**4**	33 (13.0%)	35 (13.6%)	41 (16.0%)	36 (14.1%)	31 (12.0%)	36 (14.0%)	24 (9.5%)
**5**	24 (9.5%)	27 (10.5%)	35 (13.7%)	26 (10.2%)	37 (14.3%)	25 (9.7%)	18 (7.1%)
**6**	33 (13.0%)	33 (12.8%)	34 (13.3%)	17 (6.7%)	30 (11.6%)	21 (8.1%)	7 (2.8%)
**7**	32 (12.6%)	34 (13.2%)	25 (9.8%)	11 (4.3%)	23 (8.9%)	5 (1.9%)	2 (0.8%)
**8**	36 (14.2%)	33 (12.8%)	14 (5.5%)	5 (2.0%)	10 (3.9%)	1 (0.4%)	1 (0.4%)

**9**	20 (7.9%)	22 (8.5%)	9 (3.5%)	2 (0.8%)	2 (0.8%)	1 (0.4%)	0
**10**	10 (4.0%)	5 (1.9%)	0	0			

Participants are grouped according to the count of serotypes for which they meet the corresponding condition (at least 2 or 4-fold increase from baseline) at a given visit. The group may vary from one visit to another. MOPA O8 serotype is not included, N, number of participants; ECL, electrochemiluminescent-based assay; MOPA, multiplex opsonophagocytic assay.

Supplemental Fig. 1 Number of solicited local and systemic adverse events over time.

a Solicited local adverse events

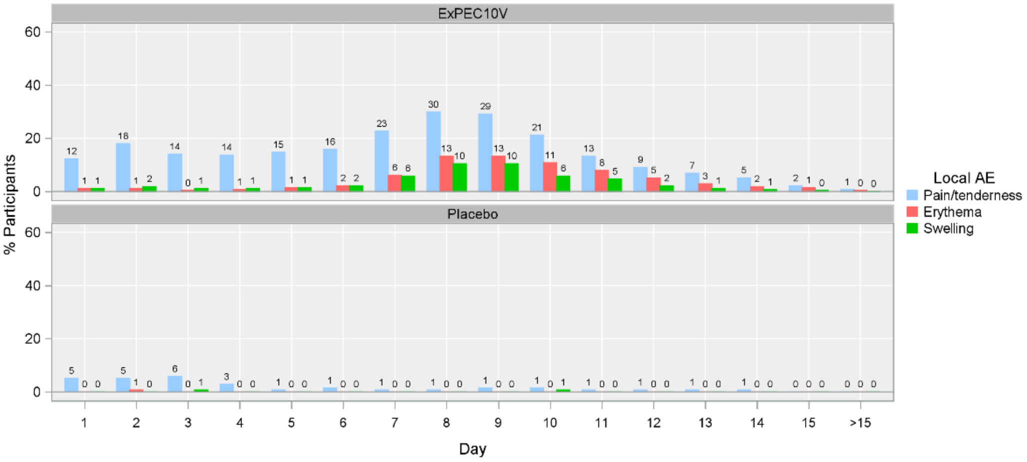

b Solicited systemic adverse events

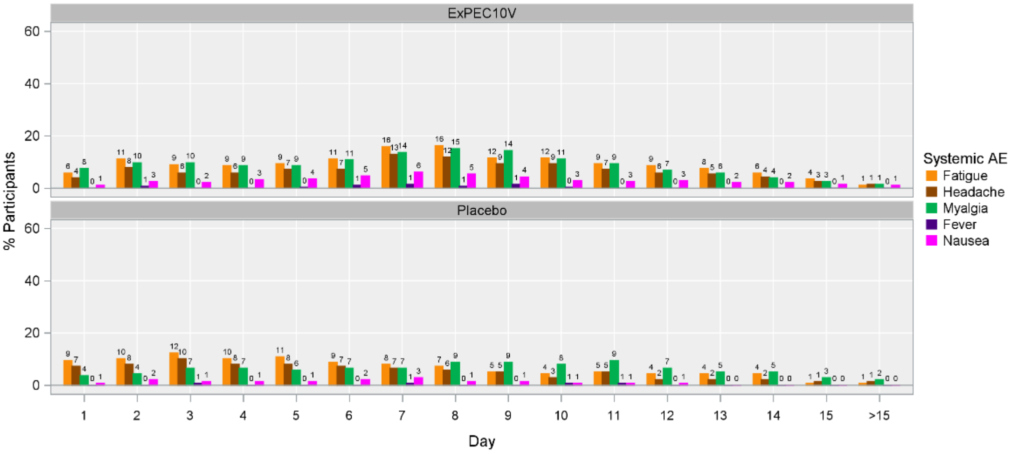

AE, adverse event.
